# A Rare Case of Transient Second-Degree Mobitz Type II Heart Block Complicating a Saddle Pulmonary Embolism

**DOI:** 10.7759/cureus.34329

**Published:** 2023-01-29

**Authors:** Muhammad Ghallab, Lilian Tran, Ibrahim Shahid, Salma Abdelmoteleb, Ibrahim Mohamed, Allison Foster, Zakaria Alagha, Most Munira

**Affiliations:** 1 Internal Medicine, Icahn School of Medicine at Mount Sinai, New York, USA; 2 Osteopathic Medicine, New York Institute of Technology, New York, USA; 3 Internal Medicine, The New York Institute of Technology College of Osteopathic Medicine, New York, USA; 4 Internal Medicine, Cairo University School of Medicine, Cairo, EGY; 5 Internal Medicine, Cairo University, Cairo, EGY; 6 Internal Medicine, Marshall University Joan C. Edwards School of Medicine, Huntington, USA; 7 Cardilogy and Medicine, Icahn School of Medicine at Mount Sinai, New York, USA; 8 Cardiology, Queens Hospital Center, New York, USA

**Keywords:** right bundle branch block, mobitz type 2 av block, second degree heart block, massive pulmonary embolism, pulmonary embolism

## Abstract

Presentations of pulmonary embolism (PE) are often associated with various cardiac arrhythmias and conduction abnormalities detected on electrocardiograms (EKG). We describe a 65-year-old female with no known history of heart disease or arrhythmias who presented with an acute onset of shortness of breath. Initial EKG showed right bundle branch block (RBBB), and first-degree atrioventricular (AV) block with subsequent development of second-degree Mobitz type II AV block. The patient’s clinical appearance was highly suggestive of a massive pulmonary embolism with hemodynamic instability, and treatment with alteplase (tPA) was given, followed by heparinization. A CT pulmonary angiography confirmed the provisional diagnosis and revealed a large saddle embolus within the right and left main pulmonary arteries. Subsequent EKG showed resolution of the RBBB, first-degree AV block, and second-degree AV block. The patient improved clinically and was discharged to a subacute rehab facility with follow-up appointments. This case highlights that pulmonary embolism may present with many EKG changes, including RBBB, first-degree, second-degree, or complete heart block. Early recognition of PE and thrombolytic treatment can improve cardiac function and restore heart rhythms. Further evaluation for underlying conduction abnormalities can later be performed.

## Introduction

The pathophysiologic changes due to pulmonary emboli (PE) with resulting cardiac strain or ischemia can be seen as acute changes in the electrocardiogram (EKG) with variable EKG changes [1,2]. The hemodynamic effect of pulmonary embolism is variable in each case and depends on several factors, including the underlying cardiac or pulmonary risk factors, the size of the embolus, or neuroendocrine factors [3]. We describe a case of first-degree heart block, second-degree Mobitz type II atrioventricular (AV) block, and right bundle branch block (RBBB) secondary to a large saddle PE. This patient’s hospital course was further complicated by hemodynamic compromise. Early treatment of shock and PE proved efficacious in both clinical conditions and the resolution of acute electrocardiographic changes. 

## Case presentation

A 65-year-old female with a past medical history of hypertension was brought to our hospital for acute onset shortness of breath. Upon arrival, the patient could not speak in complete sentences and had increased work of breathing and tachypnea. Vital signs showed tachycardia in the 110s-130s, hypoxia with oxygen saturation 60% to 70% on a 15 L/min non-rebreather mask, and hypotension with a blood pressure of 77/63 mmHg. She was placed on noninvasive ventilation bilevel positive airway pressure (BIPAP) mask for hypercapnia respiratory failure without significant improvement in her oxygen saturation and thus was intubated and mechanically ventilated. 

**Figure 1:**
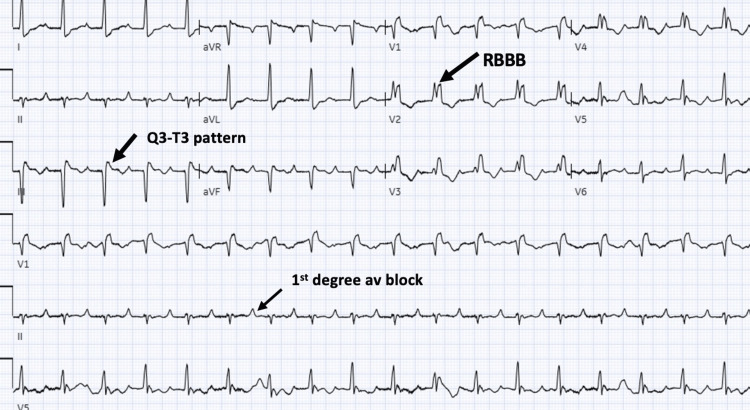
The 12-lead EKG shows sinus tachycardia at 107 beats per minute, first-degree heart block, right bundle branch block, and S1-Q3-T3 pattern. EKG: Electrocardiogram

Initial EKG showed sinus tachycardia at 107 beats per minute, first-degree heart block, RBBB, and S1-Q3-T3 pattern (Figure 1). A bedside sonogram showed a dilated right ventricle with left ventricular compression and an enlarged inferior vena cava (IVC) with a hyperechoic density, suggestive of a clot. 

Initial workup was significant for respiratory acidosis, hypercapnia, hypoxia, hypokalemia, elevated D-dimer, elevated troponin-I, and elevated lactate. The rest of the blood work was within the normal range (Table 1).

**Table 1:** Initial blood work following the patient's hospitalization ALT: Alanine transaminase, AST: Aspartate aminotransferase, PO2: Partial pressure of oxygen, PCO2: Partial pressure of carbon dioxide

Labs	Value	Reference range
Complete blood count		
Hemoglobin (Hb)	11.5 g/dl	12.0-16.0 g/dL
White blood count (WBC)	5.26 x 10(3)/mcL	4.8-10.8 x 10(3)/mcL
Platelets	274 x 10(3)/mcL	150-450 x 10(3)/mcL
Kidney functions tests		
Blood urea nitrogen	6 mg/dL	6-23 mg/dL
Creatinine	0.71 mg/dL	0.5-1.2 mg/dL
Sodium	143 mmol/L	136-145 mmol/L
Potassium	3.0 mmol/L	3.5-5.1 mmol/L
Liver function tests		
ALT	35 U/L	0-33 U/L
AST	38 U/L	5-32 U/L
Coagulation profile		
D-dimer	7,853 ng/mL	≤ 285 ng/mL
Activated partial thromboplastin time (aPTT)	28.0 seconds	25.1 - 36.5 seconds
Prothrombin time (PT)	13.7 seconds	10.0 - 13.0 seconds
Arterial blood gases (ABG)		
PH	7.05	7.35-7.45
PO2	48 mmHg	83-108 mmHg
PCO2	69 mmHg	32-35 mmHg
Lactate	8.2 mmol/L	0.4-0.8 mmol/L
Troponin-I	0.121 ng/ml	≤ 0.010 ng.ml
Thyroid-stimulating hormone (TSH)	1.77 uIU/mL	0.27 - 4.20 uIU/mL

**Figure 2:**
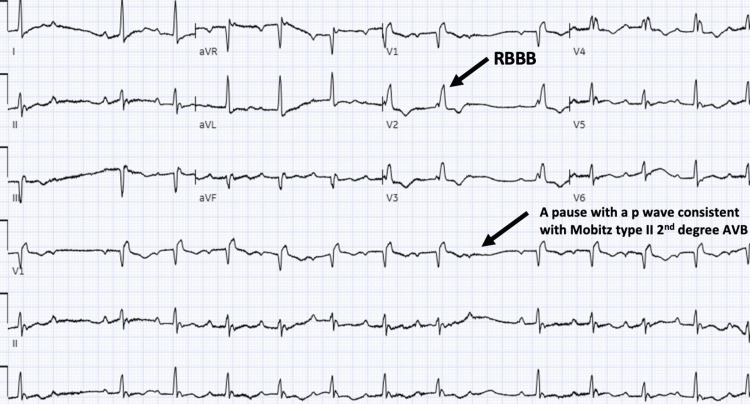
The 12-lead EKG shows sinus rhythm at 70 bpm, intermittent second-degree Mobitz type II AV block, and right bundle branch block EKG: Electrocardiogram

After intubation, the patient had an episode of bradycardia in which ECG showed sinus rhythm at 70 bpm, intermittent second-degree Mobitz type II AV block, and RBBB (Figure 2).

**Figure 3:**
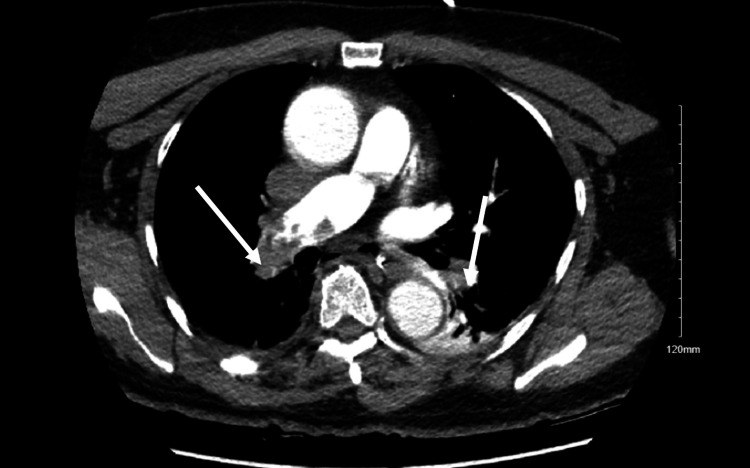
The CT pulmonary angiography shows bilateral filling defects within the pulmonary arterial tree, indicating extensive bilateral pulmonary emboli

The CT pulmonary angiography was initially deferred due to hemodynamic instability. The patient's clinical data were highly suggestive of a massive PE with hemodynamic instability, and tPA was given, followed by IV heparin infusion. Once hemodynamically stable, CT pulmonary angiography revealed acute pulmonary emboli bilaterally within the pulmonary arterial tree, including a large saddle embolus within the right and left main pulmonary arteries (Figure 3). 

**Figure 4:**
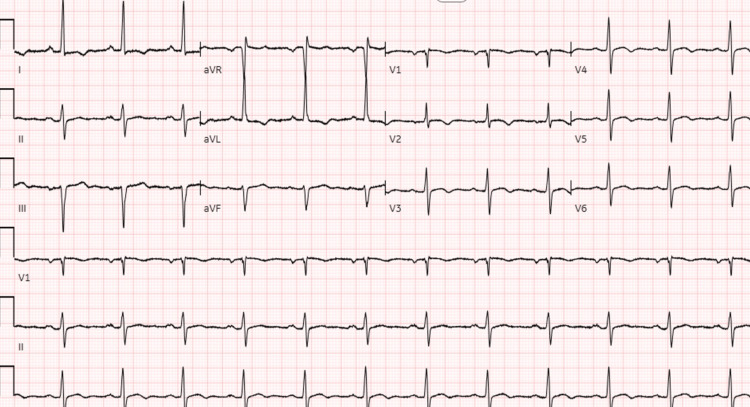
The 12-lead EKG shows normal sinus rhythm at a rate of 75 bpm and left ventricular hypertrophy with secondary T wave inversions in the lateral leads. Note the resolution of the right bundle branch block, first-degree AV block, and second-degree AV block. AV: Atrioventricular

Trans-thoracic echocardiogram (TTE) revealed dilated right ventricle, right ventricular strain, and left ventricular apical hypertrophy. The patient was placed on vasopressors (noradrenaline) and slowly tapered down. An interventional radiology (IR)-guided balloon maceration and direct thrombolysis were performed, followed by heparin infusion that was transitioned to apixaban. Subsequent EKG three days after alteplase injection showed the resolution of the RBBB, first-degree AV block, and second-degree Mobitz type II block (Figure 4). A repeat TTE showed improved right ventricular function and size, and the patient was extubated the following day. A lower limb venous duplex revealed a thrombus within the right popliteal vein, most likely the PE source. 

The patient improved clinically and was discharged to a subacute rehab facility because of physical deconditioning. During the follow-up with the hematology clinic, she was advised to take the apixaban for life. During her follow-up at the cardiology clinic, she reported having shortness of breath with exercise and going up steps but denied any chest pain. The EKG showed sinus rhythm at 73 bpm and left ventricular hypertrophy, with no ST or T wave changes. The patient was placed on a 24-hour Holter monitor that showed periods of sinus arrhythmia and blocked atrial premature contractions; otherwise, it was predominantly sinus rhythm. 

## Discussion

Our case describes a patient with no known history of arrhythmias or heart disease presenting with a PE that led to acute changes in her cardiopulmonary physiology resulting in hemodynamic collapse. In PE, the pulmonary artery obstruction leads to a rise in the right ventricular pressures and a volume overload state [4]. With this volume expansion, the right ventricle may compress the interventricular septum leading to a new RBBB on the EKG. As seen on our patient's TTE, she had a dilated right ventricle that may have led to compression of her left ventricle and thus, hemodynamic compromise. 

Patients with acute PE may have EKG findings of cor-pulmonale, including sinus tachycardia, RBBB, right axis deviation, T wave inversions, ST segment depressions, ST segment elevations, and S1Q3T3 [5,6]. The incidence of an RBBB in association with PE ranges from 6% to 69%. An RBBB is a sign of acute RV strain [7]. The mechanism behind an RBBB finding may be related to the dilation of the RV. Since the right bundle branch runs superficially along the right ventricular side of the interventricular septum, any acute distension of the right ventricular cavity can lead to changes seen on EKG [6]. An RBBB pattern is more frequent in cases of massive trunk obstruction than peripheral embolism due to RV overload, as seen in our patient [7]. Prior history of damage to the right bundle branch, as seen in anterior myocardial ischemia, which shares the same blood supply, can also explain the development of an RBBB during hypoxia or cardiac strain [5].

In addition to the RBBB, our patient developed first-degree heart block and Mobitz type II second-degree heart block. There are a few mechanisms discussed in prior cases regarding how the heart block develops in patients who develop PE. First, a massive embolism could trigger transient myocardial ischemia to the AV node resulting in a heart block [6]. Our patient's AV block and the RBBB subsided on subsequent EKG following initial therapy, suggesting the block was related to acute PE. 

An alternatively proposed mechanism is that PE may trigger a high parasympathetic drive resulting in stimulation of the Bezold-Jarish reflex [6]. This reflex is thought to be cardioprotective in response to noxious ventricular stimuli sensed by both chemoreceptors and mechanoreceptors within the left ventricular wall [8]. It consists of a triad of hypotension, bradycardia, and coronary artery dilation [9]. The activated receptors increase parasympathetic tone and decrease sympathetic tone, possibly leading to the formation of an AV block [6]. This mechanism is thought to occur in patients with prior diseased conduction systems. Our patient does not have any prior EKGs or echocardiograms for further analysis of this mechanism. 

Treatment of these cases is done on a case-by-case basis. Treatment of PE itself involves the use of therapeutic anticoagulation. Treatment of the arrhythmia depends on what type of heart block it is i.e., incomplete versus complete. Sometimes, it can involve a temporary pacemaker placement while treating the PE. After the resolution of the PE, further evaluation of the cardiac rhythm is performed to determine further management of the electrophysiological (EP) disease. If the patient is still in complete heart block post-treatment of PE, a long-term pacemaker device can be placed. If the heart block is resolved, then an EP study can be done to evaluate whether or not the patient requires or will benefit from a long-term pacemaker [4]. 

In our patient's case, she had a saddle PE, first-degree heart block, Mobitz type II second-degree heart block, and RBBB, which resolved from thrombolytic therapy with no long-standing EKG changes. Other cases similarly found resolution of complete heart block after treatment of PE [10,11]. Our patient had no prior history of conduction system disease, which may be a factor in the full resolution of the AV block caused by PE. Other authors have proposed that more severe third-degree heart block related to PE, or the sub-total resolution of blocks after treatment of PE, may suggest a prior history of conduction abnormalities [6].

## Conclusions

Pulmonary embolism may present with various EKG changes, including RBBB, first-degree, second-degree, or complete heart block. If there is high suspicion for PE based on clinical presentation, treatment with thrombolytics should be started even if CT cannot be performed due to hemodynamic instability. Early recognition of PE and thrombolytic treatment can improve cardiac function and restore heart rhythms. Further evaluation for underlying conduction abnormalities can later be performed. 
